# Insights on Molecular Mechanisms of Ovarian Development in Decapod Crustacea: Focus on Vitellogenesis-Stimulating Factors and Pathways

**DOI:** 10.3389/fendo.2020.577925

**Published:** 2020-10-06

**Authors:** Vidya Jayasankar, Sherly Tomy, Marcy N. Wilder

**Affiliations:** ^1^Marine Biotechnology Division, Madras Research Centre, ICAR-Central Marine Fisheries Research Institute, Chennai, India; ^2^Genetics and Biotechnology Unit, ICAR-Central Institute of Brackishwater Aquaculture, Chennai, India; ^3^Fisheries Division, Japan International Research Center for Agricultural Sciences, Tsukuba, Japan

**Keywords:** commercial aquaculture, crustacea, methyl farmesoate, red pigment-concentrating hormone, serotonin, vitellogenesis, vitellogenesis-stimulating hormone

## Abstract

Vitellogenesis in crustaceans is an energy-consuming process. Though the underlying mechanisms of ovarian maturation in decapod Crustacea are still unclear, evidence indicates the process to be regulated by antagonistically-acting inhibitory and stimulating factors specifically originating from X-organ/sinus gland (XO/SG) complex. Among the reported neuromediators, neuropeptides belonging to the crustacean hyperglycemic hormone (CHH)-family have been studied extensively. The structure and dynamics of inhibitory action of vitellogenesis-inhibiting hormone (VIH) on vitellogenesis have been demonstrated in several species. Similarly, the stimulatory effects of other neuropeptides of the CHH-family on crustacean vitellogenesis have also been validated. Advancement in transcriptomic sequencing and comparative genome analysis has led to the discovery of a large number of neuromediators, peptides, and putative peptide receptors having pleiotropic and novel functions in decapod reproduction. Furthermore, differing research strategies have indicated that neurotransmitters and steroid hormones play an integrative role by stimulating neuropeptide secretion, thus demonstrating the complex intertwining of regulatory factors in reproduction. However, the molecular mechanisms by which the combinatorial effect of eyestalk hormones, neuromediators and other factors coordinate to regulate ovarian maturation remain elusive. These multifunctional substances are speculated to control ovarian maturation possibly via the autocrine/paracrine pathway by acting directly on the gonads or by indirectly exerting their stimulatory effects by triggering the release of a putative gonad stimulating factor from the thoracic ganglion. Acting through receptors, they possibly affect levels of cyclic nucleotides (cAMP and cGMP) and Ca^2+^ in target tissues leading to the regulation of vitellogenesis. The “stimulatory paradox” effect of eyestalk ablation on ovarian maturation continues to be exploited in commercial aquaculture operations, and is outweighed by the detrimental physiological effects of this procedure. In this regard, the development of efficient alternatives to eyestalk ablation based on scientific knowledge is a necessity. In this article, we focus principally on the signaling pathways of positive neuromediators and other factors regulating crustacean reproduction, providing an overview of their proposed receptor-mediated stimulatory mechanisms, intracellular signaling, and probable interaction with other hormonal signals. Finally, we provide insight into future research directions on crustacean reproduction as well as potential applications of such research to aquaculture technology development.

## Introduction

Female reproduction in decapod crustaceans is a complex and precisely regulated biological process controlled by an elaborate endocrine system. Vitellogenesis, the production and accumulation of vitellin in developing oocytes, is crucial to ovarian maturation (1–3) and relies on the coordinated actions of environmental cues and hormones along the hypothalamic-pituitary-gonadal axis in vertebrates (4, 5). Analogous to the vertebrate hypothalamic/neurohypophyseal system, in crustaceans, a neurosecretory tissue in the eyestalks, the X-organ/sinus gland (XO/SG) complex, along with the central nervous system, release several neuropeptides that exert responses in other distant target tissues (6–8). Though the antagonistic and multi-interlinked neuroendocrine cascades are not as well understood in crustaceans as in vertebrates, a bi-hormonal signaling axis consisting of neuropeptides [negatively-acting vitellogenesis-inhibiting hormone (VIH), also known as gonad-inhibiting hormone (GIH) and the putative vitellogenesis-stimulating hormone (VSH)] is considered to exist. Such substances are thought to be synthesized and released into the circulation, and may then bind G protein-coupled receptors (GPCRs) on target cell surfaces, thus activating downstream cascades as a central facet of crustacean reproductive endocrinology (1, 9, 10).

Among the known neuroendocrine factors regulating crustacean reproduction, the eyestalk neuropeptides of the crustacean hyperglycemic hormone (CHH)-family encoding multi-genes (crustacean hyperglycemic hormone (CHH), gonad/vitellogenesis-inhibiting hormone (GIH/VIH), molt-inhibiting hormone (MIH), and mandibular organ inhibiting hormone (MOIH)) have been studied intensively for their broad-spectrum roles (9–12). Amongst the CHH-family peptides, VIH has received much attention for its inhibitory effects on vitellogenesis in crustaceans whereby it suppresses vitellogenin production in the target tissues or inhibits protein uptake by the oocytes (13, 14). In the whiteleg shrimp, *Litopenaeus vannamei*, levels of VIH in the hemolymph were lower during vitellogenesis than at the immature and previtellogenic stages, in agreement with its inhibitory role in vitellogenesis (15). Silencing of VIH gene expression (16–18) and injection of VIH recombinant protein (19) has further provided confirmatory evidence for its inhibitory roles on ovary development. Other members of the CHH-family are reported to have various functions; the regulation of carbohydrate metabolism, inhibition of ecdysteroid synthesis at the Y-organs, and the suppression of methyl farnesoate synthesis at the mandibular organs are attributed to CHH, MIH, and MOIH, respectively (9–12). However, these substances also play crucial roles in regulating maturation in crustaceans. Isoforms of CHH and MIH were reported to have a stimulatory effect on the growth of oocytes and vitellogenesis in the American lobster, *Homarus americanus* and in *L. vannamei* (20, 21). The use of recombinant protein and RNAi has been useful in elucidating the roles of eyestalk hormones; for example, CHH was shown to regulate the gene expression of insulin-like androgenic gland hormone (IAG) in male *L. vannamei* (22) and such methodology provided supporting evidence for a stimulatory function of MIH in reproduction (23). In addition, several studies have proposed the existence of a gonad-stimulating factor, the so-called VSH, in the brain and thoracic ganglia of crustaceans, although its identity is still unknown.

Unilateral eyestalk ablation, a commonly practiced technique used to manipulate the endocrine system in order to stimulate gonadal maturation and spawning in captivity, is based on the assumption that eyestalk removal diminishes VIH production. However, this destructive technique alters the physiology of the animal, resulting in its offspring, or seed, having inferior quality and being of less quantity, with subsequent reduced performance and death of the parent broodstock (24). Consequently, developing alternative techniques to eyestalk ablation for controlling ovarian maturation in captivity is challenging, and attempts at resolving such issues have focused on the administration of exogenous compounds to stimulate vitellogenesis (25–29), and to reduce circulatory levels of inhibitory hormones via the use of VIH antibodies and RNA interference (RNAi) to achieve host-induced gene silencing of VIH (17–19).

**Figure 1:**
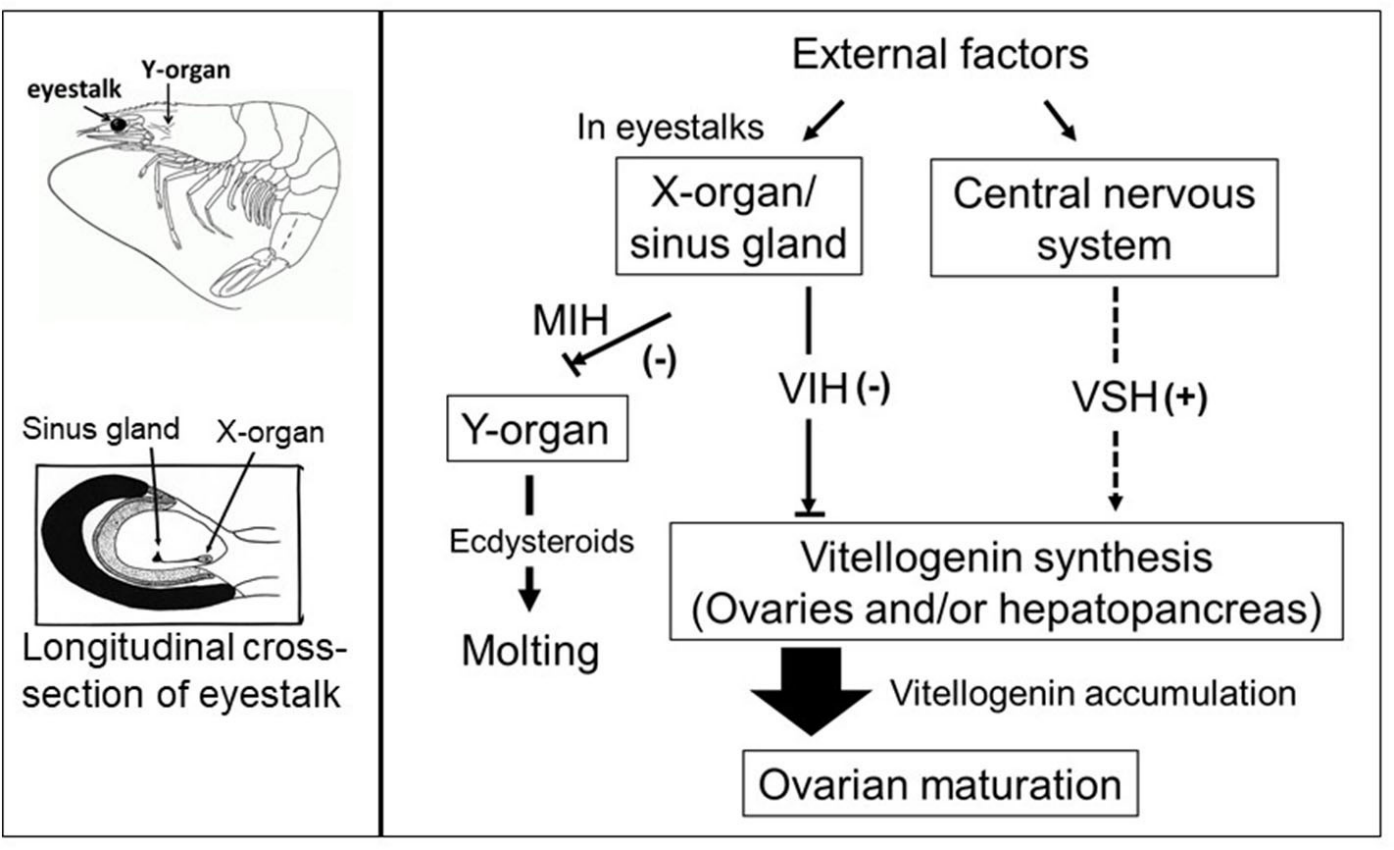
Simplified schematic diagram of the control of molting and reproduction in decapod Crustacea (right-hand side). External factors such as water temperature, salinity, and pressure, season and daylength, and availability of nutrition, are thought to ultimately influence the X-organ/sinus gland (XO/SG) complex and central nervous system. It is well-established that the XO/SG complex is the source of vitellogenesis-inhibiting hormone (VIH) and molt-inhibiting hormone (MIH). In contrast, there are many potential pathways that fulfill the role of putative vitellogenesis-stimulating hormone (VSH) as described in the text. The locations of the eyestalks and Y-organs are indicated using a generic drawing of a shrimp (top left), and a close-up representation of the eyestalk is also presented (bottom left).

In addition to eyestalk peptides, the neuroendocrine regulation of crustacean reproduction involves the interactions of several auxiliary factors including biogenic amine neurotransmitters (7, 30), as well as ecdysteroids (31) and the sesquiterpenoid methyl farnesoate (MF) (32) which are synthesized and released by the Y-organ and the mandibular organs, respectively. Thus, an intricate network, consisting of an array of chemically diverse molecules, harmonizes to establish a signaling cascade regulating gonad development in crustaceans. However, the molecular mechanisms by which these multifunctional neuromediators and other factors are involved in the upstream control of neuropeptide hormone release from the XO/SG complex, as well as from other neuroendocrine organs and secretory neurons, remains elusive. A simplified schematic diagram of the control of molting and reproduction in decapod Crustacea is shown in Figure 1. Various external factors related to water temperature, salinity, and pressure, season and daylength, and availability of nutrition, are thought to ultimately influence the X-organ/sinus gland (XO/SG) complex and central nervous system. This figure has been abbreviated to show only the involvement of VIH and putative VSH; it is on the basis of this concept that eyestalk ablation is used in commercial hatcheries in order to diminish VIH levels and induce maturation/spawning.

More recently, advances in *in silico* neuropeptidome research and comparative genome analysis have led to the discovery of a large number of decapod hormones and neuropeptide-encoding transcripts in the crustacean eyestalk, cerebral ganglia, and also in extra-neural tissues (namely heart, midgut, gills, hepatopancreas, muscle, stomach, and ovaries) with pleiotropic and novel functions including reproduction in several crustacean species (33–35). Research findings also suggest that these neuropeptides harbor autocrine and/or paracrine functions, although the roles of many of them in crustaceans are yet unknown (9, 33, 35). Additionally, although a publicly available complete decapod genome is now available for *L. vannamei* (36) and the marbled crayfish, *Procambarus virginalis* (37), it remains difficult to interpret the roles of the majority of novel neuropeptides identified in crustaceans. Nevertheless, such information will be of help in interpreting the putative roles of identified novel neuropeptides. Therefore, in consideration of the above, while the negatively-controlled facets of reproduction are fairly-well understood, the stimulatory side of reproductive control offers scope for further research. This review therefore focuses on the possible signaling pathways of non-CHH-family neuromediators and other factors that are thought to promote gonadotrophic activity in female decapod crustaceans, with the aim of clarifying what is currently known, and possibly suggesting a means of developing viable technology for the control of female maturation in captivity. We thus provide an overview of reported receptor-mediated stimulatory effects of such substances, intracellular signaling, and their synergy with other hormonal signals, in order to underline both their known and putative roles in crustacean reproduction.

## Serotonin

### Serotonergic Pathways Regulate Ovarian Maturation in Crustaceans

The regulatory role of neurotransmitters in crustacean reproduction is well-documented (38). Among the identified major neurotransmitters in crustaceans (serotonin, dopamine, melatonin, and octopamine), serotonin (also referred to as 5-hydroxytryptamine, 5-HT), a biogenic amine derived from the amino acid tryptophan, has been reported to play a prominent role in crustacean reproduction. It is ubiquitously found in several microorganisms, and throughout the plant and animal world in significant amounts; it functions as a neurotransmitter in the brain and as a neurohormone in the periphery, regulating several important physiological functions both in vertebrates and invertebrates. In invertebrates, serotonin modulates multiple functions including circadian rhythms (39), neurogenesis (40), osmotic adjustment (41), growth (42), aggression (43), molting (44), and reproduction (27). Several reports have correlated the role of serotonin in the regulation of growth and reproductive development of vertebrates, rather than focusing on invertebrates. In fishes, it has been reported as a regulator of follicular growth (45). In invertebrates, the serotonin system plays a unique role in the initiation and maintenance of reproductive function.

Emerging evidence suggests serotonin to be the upstream neurotransmitter controlling reproduction by acting at the eyestalks and the central nervous system in crustaceans (7, 30, 46, 47). Serotonergic neuromodulation of reproductive function resulted in a significant increase in oocyte diameter and enhanced ovarian maturation in *P. indicus* (27, 48), *Litopenaeus vannamei* (49), *Penaeus monodon* (50), *Fenneropenaeus merguiensis* (51), *Macrobrachium rosenbergii* (46, 52), and *Procambarus clarkii* (53). Injection of serotonin to the giant freshwater prawn *M. rosenbergii*, shortened the normal duration required for ovarian maturation in females and brought about a significant increase in the testis-somatic index in males (54). Furthermore, induced precocious reproduction was reported in the freshwater edible crab *Oziothelphusa senex senex* following administration of serotonin (55). Increasing levels of serotonin were observed in the ovaries of *P. monodon* (50), *L. vannamei* (56), and *M. rosenbergii* (52) during the early ovarian maturation stages, reaching maximum levels at the mature ovarian stages. In support of this observation, Soonthornsumrith et al. (57) also suggested that serotonin in both the central nervous system and ovary act in harmony to control oocyte maturation. A combination of spiperone (a dopamine antagonist) and serotonin was shown to induce ovarian maturation in *M. rosenbergii* (58) together with enhanced spawning in *L. vannamei* and *Litopenaeus stylirostris* (59) compared with the injection of serotonin alone. Serotonin also stimulated male reproductive parameters and testis development in the narrow clawed crayfish, *Pontastacus leptodactylu*s (60) and the crayfish *P. clarkii* (61).

The endocrine pathway where serotonin is considered to stimulate downstream reproductive hormones is hypothesized to be performed mainly by the action of serotonin at the eyestalk and/or the central nervous system level. In more detail, serotonin may function to regulate the synthesis and release of endocrine factors such as VSH and/or VIH, or may also act upon red pigment-concentrating hormone (RPCH) to serve as an intermediary substance, causing its release from eyestalk neural tissue, which may, in turn, stimulate the release of the putative VSH (27, 38, 46, 47). A faster response in ovarian maturation observed in ablated females of *P. indicus and F. merguiensis* treated with serotonin suggests that lowering levels of inhibitory hormones is an essential prerequisite for serotonin to effectively induce maturation (27, 62). Tomy et al. (27) provided confirmatory evidence for serotonin-induced ovarian development to be significant but less effective than eyestalk ablation. Meeratana et al. (46) reported that serotonin-primed thoracic ganglion medium accelerated ovarian development in *M. rosenbergii*, indicating an indirect effect of serotonin on reproduction. The localization of serotonin-immunoreactive cells in the X-organ neurons and fibers innervating the sinus gland tissue and in other parts of the central nervous system of various decapod crustaceans (57, 63) support the suggested regulatory role of serotonin in the synthesis and release of other neurohormones from the XO/SG complex such as CHH (64, 65) and probably other neuropeptides of the CHH-family (56). However, the presence of serotonin immunoreactivity was also observed in crustacean gonads (46, 56, 57, 63) and its stimulatory effects on gonad maturation confirmed, thereby further advocating an autocrine/paracrine mode of receptor-mediated action for serotonin in the control of crustacean oocyte maturation.

In crustaceans, the serotonergic system constitutes a distinct signaling system that probably acts synergistically with other stimulatory factors forming an overarching system that regulates vitellogenesis and oocyte maturation. Contrary to the negative regulation of vitellogenesis performed by the CHH-family peptide VIH, serotonin is reported to regulate in a stimulatory manner, directly or indirectly, the action of other hormones and reproductive-related proteins in crustaceans such as RPCH (66), MIH (67), tachykinin and neuropeptide F (NPF; the invertebrate equivalent of neuropeptide Y) (68), and farnesoic acid O-methyltransferase (FAMeT) (29). Sathyanandam et al. (64) reported that serotonin injection resulted in hyperglycemia by triggering the release of CHH in *P. indicus*. A recent report by Soonthornsumrith et al. (57) indicated that serotonin enhanced the secretion of ovarian steroids (estradiol and progesterone) in mature ovarian explants from *M. rosenbergii* and suggested that serotonin-regulated ovarian maturation via the induction of female sex steroid hormone release in turn stimulated vitellogenesis.

Girish et al. (69) proposed that serotonin treatment increases the gene expression levels of retinoid X receptor (RXR) and ecdysteroid receptor (EcR) in the hepatopancreas and ovary of *Scylla serrata*, thereby upregulating methyl farnesoate and ecdysteroid synthesis, respectively. Furthermore, the upregulation of three reproductive-related genes, namely those of farnesoic acid O-methyltransferase (*FAMeT*), estrogen sulfotransferase (*ESULT*) and prostaglandin F synthase (*PGFS*) was evidenced by transcriptome data from the nervous tissue of female mud crab (*Scylla olivacea*) injected with serotonin; this is considered to provide additional supporting evidence of the stimulatory role of serotonin (47). Finally, serotonin was proposed to enhance the release of GnRH-like peptide from the nervous tissue with the result of stimulating reproduction in male *M. rosenbergii*; most likely, this occurred via a mechanism where the release of VIH from eyestalks is inhibited (70).

The serotonin system may also be modulated by reproductive hormones. In mammals, ovarian steroids such as progesterone and estrogen regulate the content of serotonin in the brain (71). Collectively, these results suggest that serotonin is a potent gonadotrophic agent in crustaceans, the actions of which are closely associated with other reproductive endocrine signaling pathways. However, there remain critical caveats in our understanding of the precise role of serotonin in crustaceans, specifically their potential interactive roles with other neurochemical systems; in this regard, a more complete holistic view of how crustacean reproduction is regulated, needs to be achieved.

### Receptor-Mediated Action of Serotonin

Serotonin coordinates several physiological processes in both vertebrates and invertebrates through differential binding with specific cell-surface receptors (G protein-coupled receptors, GPCRs or ligand-gated ion channels), that activate an intracellular second messenger cascade (including cAMP and protein kinase, PKA), to elicit a serotonergic response. RNAi-mediated gene silencing of the serotonin receptor has provided evidence for the receptor-mediated action of serotonin (72). Based on structure, signaling mechanisms, biochemical, and pharmacological properties, the vertebrate serotonin receptors have been assigned to seven receptor classes (5-HT1 to 5-HT7) consisting of six GPCRs (5HT1-5HT7), and a ligand-gated ion channel (5-HT3 receptor) (73).

The GPCRs are characterized by seven transmembrane domains, an extracellular N-terminus and the intracellular C-terminus and are associated with heterotrimeric G proteins (a polypeptide comprised of a Gα subunit which binds and hydrolyzes GTP, a Gβ and Gγ subunits), that are classified into four families (i.e., Gs, Gq, Gi/o, and G12/13) based on their specific type of Gα-subunit. The activated GPCR stimulates the dissociation of the interacting heterotrimeric G protein into a Gα subunit and a Gβγ complex, thereby coordinating the downstream signal pathway in accordance with the activation of the Gα subunit. Among the G-coupled protein serotonin receptors, 5-HT1 and 5-HT5 associate preferentially with Gαi subunit of the heterodimeric protein (Gi/o) and impede cAMP synthesis, whereas 5-HT4, 5-HT6, and 5-HT7 couple preferentially with the Gαs alpha subunit (Gs) of G protein, leading to increased cAMP production. Levels of protein kinase A (PKA), a cAMP-dependent protein kinase, enhances many functions in the cell. The Gq (Gq alpha subunit)-coupled 5-HT2 mediates the hydrolysis of inositol phosphates and cause a subsequent increase in cytosolic Ca^2+^.

Blenau and Baumann (74) reported that the sequences of various serotonin receptors are conserved among vertebrates and invertebrates, indicative of their crucial functions across species. Orthologous vertebrate serotonergic GPCRs with conserved signaling pathways have been cloned and characterized in invertebrates, including crustaceans, and have been shown to belong to three major types of vertebrate GPCRs groups (5-HT1, 5-HT2, and 5-HT7) (75). An additional receptor, MOD-1, a serotonin-gated ion channel, was found in *C. elegans* and exhibited similarities to the mammalian 5-HT3 receptor, but with differences in function (76). Qi et al. (77) reported a novel receptor in the butterfly *Pieris rapae*, that was classified into a new family of receptors designated as 5-HT8. As in vertebrates, serotonin activates different downstream signaling pathways in order to control levels of second messengers, specifically adenylate cyclase activity and cAMP production (inhibited by Gi-coupled 5-HT1-like receptors, but stimulated by Gs-coupled 5-HT7-like receptors) or phospholipase C, and subsequently increases Ca^2+^ (stimulated by Gq-coupled 5-HT2-like receptors) (75), to exert both inhibitory and excitatory effects.

### Serotonin and the Onset of Oocyte Germinal Vesicle Breakdown/Serotonergic Regulation of Oocyte Germinal Vesicle Breakdown

As a “universal” conserved principle, developing primary oocytes are arrested at prophase I in advance of the ensuing events of oocyte maturation (78). The resumption of oocyte meiotic maturation, germinal vesicle breakdown (GVBD), and final release of the mature egg from the ovary are essential processes in sexual reproduction. These events are triggered initially by substances referred to collectively as “maturation initiation hormones.” More detailed discussion is beyond the scope of this review, but such maturation initiation hormones represent a variety of molecular identities, and have been reported in a range of species with the exception of mammals. This process is further orchestrated by maturation/M-phase promoting factors (an auto-regulated complex of cyclin-dependent kinase cdk1/cdc2 and its regulatory subunit cyclin B formed during oocyte maturation) and is highly conserved among animal species across the phylogenetic spectrum, thus establishing possible marker genes of oocyte developmental competence (79). The signal transduction pathway regulating maturation initiation hormone activity and subsequent process of maturation are highly complex. In vertebrates, active GPCRs coupled to Gs (e.g., Gs alpha subunit of G protein, a GTPase that acts as a cellular signaling protein) stimulate adenylate cyclase to maintain high intracellular second messenger cAMP concentrations in oocyte; this inhibits the resumption of prophase I arrest in many animal oocytes (80). Serotonergic receptors (Gq-coupled 5-HT2) are responsible for mobilizing Ca^2+^ levels in oocytes which in turn, leads to the hydrolysis of inositol phosphate and an increase in cytosolic Ca^2+^ levels (81). The maturation initiating hormone overrides this prophase arrest to initiate meiotic resumption and maturation by activating cdc2/cyclin B in oocytes, the mechanisms of which are yet unclear.

As discussed above, although the role of maturation initiation hormones during oocyte maturation has been well-studied in a wide variety of eukaryotic organisms, little is known regarding crustacean species. Cortical rod formation and GVBD are the hallmarks of oocyte maturation in penaeid shrimps (82). In naturally maturing penaeid females, elevated expression levels of reproductive genes (*vg, cdc2, cyclin B*, and *tsp)* (e.g., thrombospondin, a major component of cortical rods in penaeid shrimps) are seen with the advancement of ovarian maturation, and are considered to be indicators of the developmental competence of the oocytes (27, 83, 84). In addition, elevated expression of serotonergic receptors in invertebrate species seen at the time of maturity is considered to indicate an underlying function in promoting oocyte maturation (85). The direct receptor-mediated action of serotonin on oocytes in marine nemertean worms was associated with variation in intracellular Ca^2+^ and cAMP levels, activation of cdc2/cyclin B in oocytes, and the stimulation of meiotic resumption and maturation (86). Similarly, serotonin rapidly stimulated meiotic resumption and GVBD of oocytes in the mud crab, *Scylla paramamosain*, through receptor-mediated signaling activity downstream of cAMP pathways (85). This negative correlation between GVBD and cAMP levels was further confirmed by forskolin treatment which significantly blocked serotonin-induced GVBD (85). In the Chinese mitten crab*, Eriocheir sinensis*, cdc2 kinase and cyclin B were highly expressed in GVBD oocytes (84). Furthermore, microRNA (miR-2 and miR-133) was proposed to regulate oocyte meiosis in *E. sinensis* by inhibiting the translation or post-translation of cyclin B during meiosis (87).

**Figure 2:**
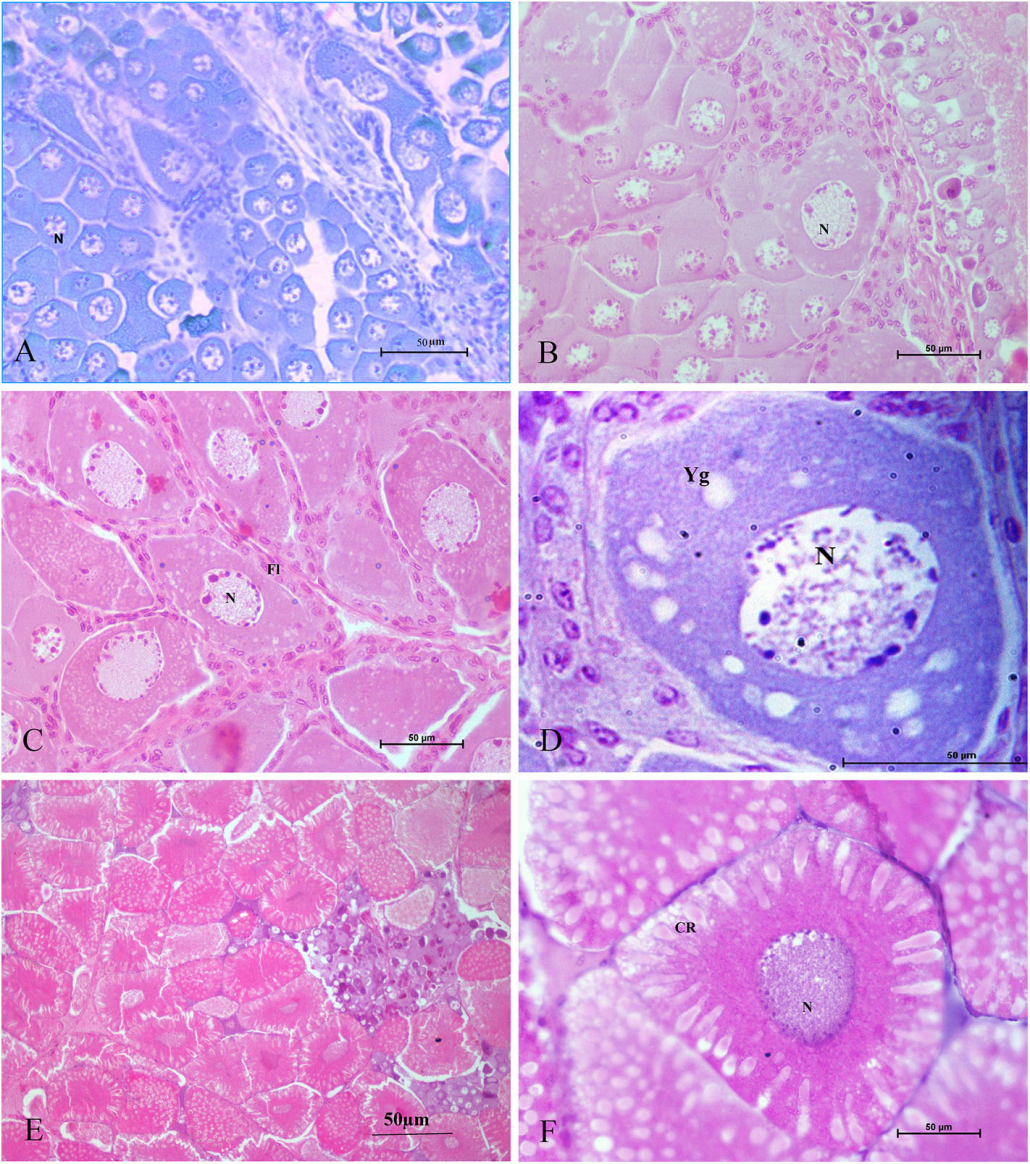
Histological sections of ovarian tissues collected from *Penaeus indicus* 14 days after treatment and stained with hematoxylin and eosin. Ovarian tissues shown are as follows: **(A)** control (10×), **(B)** serotonin group (40×), **(C)** eyestalk-ablated group in the early vitellogenic stage with numerous eosinophilic yolk granules in the peripheral ooplasm; **(D)** eyestalk-ablated group with an early vitellogenic oocyte (100×); **(E)** serotonin + eyestalk-ablated group with oocytes having club-shaped cortical rods extending the nucleus (10×) and **(F)** mature oocyte with cortical rods (40×). CR: cortical rod; Fl: follicle layer; N: nucleus; Yg: yolk granules. Reproduced with permission from Tomy et al. (27).

**Figure 3:**
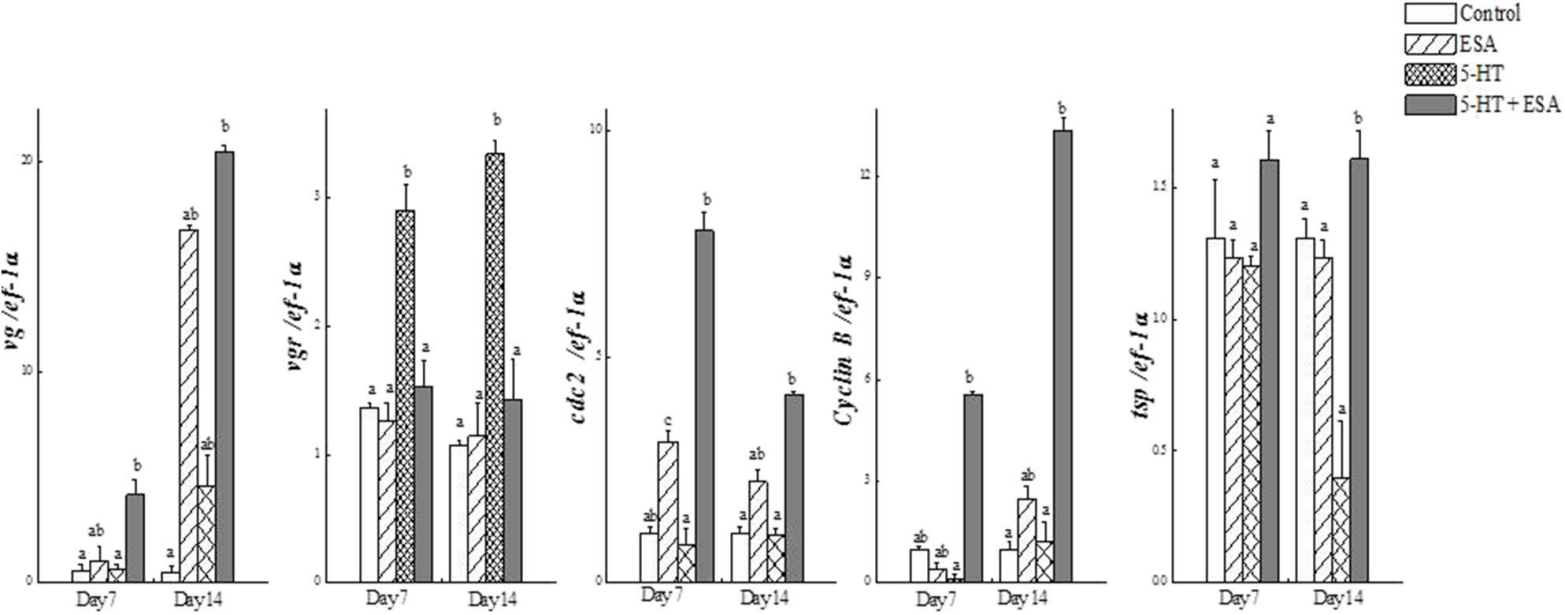
Real-time PCR analysis of (a) *vg*, (b) *vgr*, (c) *cdc2*, (d) *cyclin B* and (e) *tsp* transcripts in ovaries of *Penaeus indicus* subjected to induced maturation 7 and 14 days after treatment. Each value represents the mean ± SE (*n* = 10 shrimp). Differing letters indicate significant differences (*P* < 0.05) according to one-way ANOVA followed by Duncan's multiple range test. Reproduced with permission from Tomy et al. (27).

Tomy et al. (27) reported that the action of serotonin resulted in the resumption of oocyte meiotic maturation in Indian white shrimp, *Penaeus indicus*, by stimulating the formation of a putative maturation initiation hormone which was more significant in ablated shrimps. As shown in Figure 2, previous work by this author revealed that ablated shrimp were in the advanced stages of ovarian development as noted by the presence of numerous eosinophilic yolk granules in the peripheral ooplasm. Serotonin-treated ablated shrimps exhibited even more pronounced changes, including the presence of yolk globules in the granular cytoplasm and distinct eosinophilic club-shaped cortical rods extending toward the nucleus of elongated oocytes. In addition, the differential expression of ovarian genes involved in vitellogenesis [vitellogenin (*vg*), vitellogenin receptor (*vgr*)] and meiotic maturation [cyclin-dependent kinase 2 (*cdc2*), cyclin B and thrombospondin (*tsp*)] was shown to increase significantly in these groups, indicating that post-vitellogenic meiotic resumption and maturation of the oocytes had occurred (Figure 3). Furthermore, based on cytological and molecular evidence, the authors corroborated the stimulatory effects of serotonin on ovarian maturation in *P. indicus*, and proposed the possibility of a dual regulatory role of serotonin in both vitellogenesis and oocyte maturation in penaeid shrimps (27). However, a delayed response was observed in shrimps with intact eyestalks treated with serotonin. This substantiated the hypothesis that the stimulatory impulse of serotonin to trigger final maturation is comparatively less effective in the presence of VIH/GIH from the eyestalks. Along the lines of the above, although a great deal has been learned about the effects of serotonin on crustacean maturation, the pathways through which serotonin exerts its gonadotropic influence remains to be fully determined. Additional studies employing RNAi, recombinant protein methodology and gene editing techniques, will aid in gaining a better understanding of the positively-controlled aspects of crustacean reproductive endocrinology.

## Red Pigment-Concentrating Hormone (RPCH)

### Involvement of RPCH in Crustacean Reproduction

A related, but distinct, peptide signaling system in crustaceans is constituted by red pigment-concentrating hormone (RPCH). It was the first invertebrate neuropeptide to be fully characterized (from the eyestalks of *Pandalus borealis*) (88) and is involved in the distribution of pigments in response to environment (89) and light-dark adaptation (90). The structural similarity among RPCH, adipokinetic hormone (AKH), corazonin (Crz), adipokinetic hormone/corazonin-related peptide (ACP) and gonadotropin-releasing hormone (GnRH) in various animals resulted in clustering them together into the GnRH-superfamily (91). In decapod crustaceans, RPCH is synthesized in the XO/SG complex of the eyestalks, but is also expressed in all major parts of the nervous system, where it is suggested to act as a neurotransmitter having dual endocrine and autocrine/paracrine function (66, 92, 93).

Based on its amino acid composition and chromatographic characteristics, the primary structure of mature RPCH as sequenced from several decapod crustaceans is known to be conserved; the molecule exhibits the same octapeptide sequence (pQLNFSPGWamide), blocked N-(pyroglutamate) and C-termini (carboxyamide) features, and presence of aromatic amino acids at positions 4 (phenylalanine) and 8 (tryptophan) in various species (66, 94). Such a blocked ligand was reported to be less susceptible to the action of exopeptidases in the hemolymph insects, thus necessitating a longer half-life of the peptide to achieve its hormonal effects (94). Recently, the existence of a variant isoform of RPCH (pQVNFSTSWamide) was identified in daphnids through transcriptome mining (95). Similarly, Christie (96) identified a structurally modified RPCH in the carp louse, *Argulus siamensis*.

RPCH plays multifunctional roles in crustaceans, regulating lipid and carbohydrate mobilization (97, 98), mediating circadian (39, 89), and swimmeret rhythms (99), modulating the stomatogastric nervous system in crustaceans (100) and stimulating MF production from the mandibular organs (101). Increased glucose levels could be elicited in the hemolymph of the isopod *Porcellio scaber* injected with synthetic RPCH, suggesting a novel role for this neuropeptide in carbohydrate mobilization by causing the release of crustacean hyperglycemic hormone (CHH) (102). Sathapondecha et al. (92) reported that the expression of RPCH was transiently stimulated upon hypersalinity change within 12 h in *P. monodon*, suggesting its osmoregulatory functioning. Furthermore, the study also revealed that injection of RPCH peptide increased gill Na^+^/K^+^ ATPase activity in 36–48 h after injection. Moreover, RPCH was reported to play a role in molting, probably by mediating hemolymph osmolality and ion transport enzymes during the late premolt stages.

**Figure 4:**
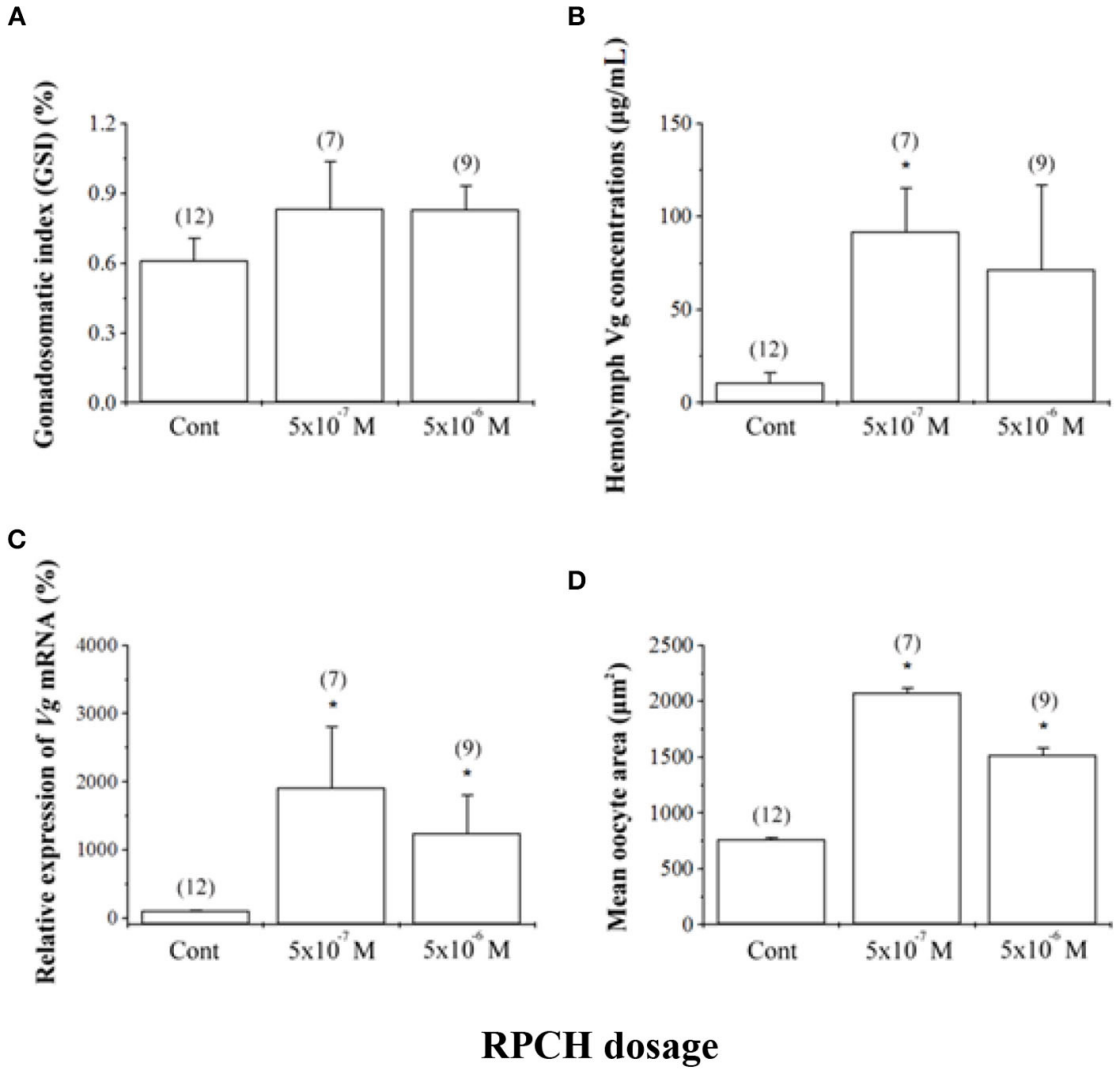
Effects of red pigment-concentrating hormone (RPCH) on ovarian growth in the whiteleg shrimp, *Litopenaeus vannamei*. Animals received repeated injections of either sterile shrimp saline (Cont) or RPCH (100 μl of 5 · 10^−7^ M or 5 · 10^−6^ M). **(A)** Gonadosomatic index (GSI), **(B)** hemolymph vitellogenin (Vg) concentrations, **(C)** relative expression of *Vg* mRNA, and **(D)** mean oocyte area. Data are shown as the mean ± SE (*n* = 7 to 12). Asterisks indicate significant differences (*P* < 0.05) with respect to the control. Reproduced with permission from Chen et al. (28).

RPCH has also been shown to have a potentially critical role in reproduction in crustaceans. RPCH transcripts were detected in ovaries and heart in addition to the neural tissue in *L. vannamei* (28), supportive of a role for this hormone in reproduction. *In vitro* co-incubation of ovary explant with nervous tissue caused significant oocyte growth when RPCH was added to the explant culture in several crustacean species (38, 103, 104). Fingerman (38) further confirmed that the combined effects of RPCH and thoracic ganglia on ovarian maturation was more significant compared to that with explants incubated with thoracic ganglia alone; this suggested that RPCH, as proposed for serotonin, acts as neurotransmitter stimulating the release of VSH, with calcium acting as a second messenger for RPCH. Contrary to the indirect role of RPCH in reproduction, Chen et al. (28) investigated the effects of synthetic RPCH on ovarian maturation in *L. vannamei* and reported higher vitellogenin mRNA expression in ovaries, increased protein levels in hemolymph, and enlarged oocyte area in treated shrimps (Figure 4); thus, a hypothesis was proposed where RPCH may have a more direct in vitellogenesis. Up-regulation of RPCH in eyestalks following serotonin-induced maturation in the whiteleg shrimp *L. vannamei* (28) and crab, *S. olivacea* (66) suggested that the downstream roles of serotonin in reproduction are mediated through RPCH. In red swamp crayfish, *P. clarkii*, RPCH promoted the synthesis and release of methyl farnesoate from the mandibular organs, which in turn regulates maturation (101). On the other hand, a significant decrease in RPCH levels in eyestalk and nervous tissue were suggested as the probable cause for ovarian degeneration in the freshwater shrimp *Macrobrachium nipponense* (105). Collectively, these findings indicate that RPCH plays a vital role in regulating reproductive functions in a similar pattern, as suggested for serotonin, via the autocrine/paracrine pathway and culminating in the release of VSH/GSH from the nervous tissues. Given the role of RPCH in reproduction, further studies utilizing different approaches such as dsRNA-mediated gene silencing or CRISPR-mediated gene editing, may lead to new insights on the role of RPCH in ovarian maturation.

### Receptor-Mediated Action of RPCH

Like other neuropeptides, RPCH exerts its effects on target cells by binding to its receptors with high affinity. Since RPCH peptides identified in decapods to date harbor identical or near-identical sequence, it is assumed that the decapod RPCH receptor (RPCHR) is also conserved in terms of its ability to bind to RPCH/AKH ligands. Buckley et al. (106) proposed the identity of a putative RPCH-like receptor to be a G-protein coupled receptor based on *in silico* mining of transcriptomic data, with this receptor being expressed during the metamorphic molt of the spiny lobster, *Sagmariasus verreauxi*. Marco et al. (107) pharmacologically characterized the red pigment-concentrating hormone receptor (RPCHR) from the water flea, *Daphnia pulex*, and revealed it to be similar in terms of sequence to insect adipokinetic hormone (AKH) receptor. This was the first report of a deorphanized neuropeptide G protein-coupled receptor (GPCR) in crustaceans. Recently, RPCHR has been deorphanized in *Carcinus maenas* and was seen to be expressed at high levels in the eyestalk neural tissue, but was also observed in the antennal gland and maturing ovary (93). The RPCHR had typical characteristics of rhodopsin-like GPCRs and interacted with RPCH with extremely high sensitivity (93).

Despite the lack of information on crustacean RPCHR sequences, investigations on the RPCH signaling cascade have been performed in several decapod crustacean species. The RPCH primarily functions in mediating pigment aggregation in crustaceans (89, 98), via the Ca^2+^-activated cGMP signaling cascade (108). Milograna et al. (109) experimentally proved that RPCH lowered cAMP levels in the ovarian chromatophores of the freshwater shrimp, *Macrobrachium olfersi*.

## Methyl Farnesoate (MF)

### Methyl Farnesoate-Mediated Ovarian Development in Crustaceans

Methyl farnesoate (MF), the major sesquiterpenoid synthesized and secreted from the mandibular organs in crustaceans, is under the negative control of MOIH from eyestalks (110). It was first characterized as a juvenile hormone-like factor in the spider crab *Libinia emarginata* (111), but was later unequivocally identified in several crustacean species. Due to the structural similarity between MF and the juvenile hormones (JH), MF is considered to be the crustacean homolog of JH. In crustaceans, farnesoic acid O-methyltransferase (FAMeT) is the rate-limiting enzyme catalyzing the methylation of farnesoic acid (FA) to MF. After release from the mandibular organs, MF is transported through the hemolymph to target tissues by MF-binding proteins (112).

MF is thought to be a key endocrine controller of several biological functions in crustaceans including molting (113), reproduction (29, 114, 115), morphogenesis (116), phenotypic plasticity (117), and osmoregulation (118). MF is capable of inducing the synthesis and release of ecdysteroids from the Y-organs (119, 120). Another significant function of MF seems to be the enhancement of reproductive maturation in both male and female crustaceans (29, 113, 114, 121, 122). A positive correlation between MF levels in hemolymph and stage of development of the ovaries was reported in *P. clarkii* (123) and in the freshwater rice field crab *O. senex senex* (124), *Portunus trituberculatus* (114), *S. paramamosain* (125), and estuarine crab *Neohelice granulate* (126), indicating a probable role in ovarian maturation in these species. Similarly, MF also stimulates testicular growth, and affects morphology and behavior in males, with MF levels being high in reproductively-active males compared to inactive males (10, 116, 121).

Elevated vitellogenin levels associated with ovarian maturation following MF administration was reported in *L. emarginata* (115), *L. vannamei* (32, 127), *P. indicus* (29), *Macrobrachium malcolmsoni* (122), *P. clarkii* (123, 128), *O. senex senex* (113), and *N. granulate* (126). MF secretion from the mandibular organs was highest during the vitellogenic stages of ovarian development (123), while MF levels were highest during the pre- and early vitellogenesis phases in crabs and shrimp (124, 127), suggesting a role for MF in relation to gonial proliferation and stimulation of vitellogenesis. In contrast, MF levels did not change with maturation in *M. rosenbergii*, but were correlated with molting (129) and MF was also demonstrated to inhibit late ovarian stage development with reduced fecundity levels in *P. monodon* (130). MF injection during the late vitellogenic phase had no significant effect on post-vitellogenesis or spawning in the freshwater crab *Travancoriana schirnerae* (131). Nevertheless, dietary inclusion of MF stimulated vitellogenesis in the crayfish *P. clarkii* and *P. monodon*, bringing about increased oocyte diameter, higher fecundity and egg fertility, and increased hatching rates (123, 132). In Daphnia, MF was suggested to act as a sex determinant (133).

The stimulatory effects of MF on ovarian maturation were observed to be pronounced in eyestalk-ablated animals, with a higher MF concentration in the hemolymph and elevated levels of FAMeT compared with eyestalk intact animals in the Indian white shrimp, *P. indicus* and the crab *O. senex senex* (29, 134). Buchi et al. (134) reported that MF caused more rapid ovarian maturation and also resulted in increased vitellogenin mRNA expression in hepatopancreas fragments incubated with MF in the crab *O. senex senex*; this suggested a direct action on vitellogenin expression at the hepatopancreas or indirect stimulation through other biologically-active molecules (such as ecdysteroids from the Y-organs). Similarly, up-regulation of vitellogenin gene expression was observed in i*n vitro* hepatopancreas explant cultures from the red crab, *Charybdis feriatus* when MF was added at higher concentrations (135). On the contrary, Tiu et al. (136) reported that hepatopancreas explants from American lobster *H. americanus* at different vitellogenic stages treated with MF did not show any significant increases in expression of the vitellogenin gene. Furthermore, evidence for the stimulatory effects of MF on vitellogenin gene expression in crustaceans was obtained from experiments on ovarian explants with MF, where the addition of MF to culture media resulted in increased vitellogenin gene expression (29). Mandibular organ explants cultured in the presence of serotonin showed no significant increase in MF secretion levels, suggesting that serotonin-mediated MF synthesis is indirect, probably based on the inhibition of MOIH release (69). There is evidence that MF, in combination with vertebrate steroid hormones like 17-hydroxyprogesterone (126) and 17β-estradiol (128), regulates vitellogenesis in crustaceans. Swetha et al. (137) reported that prostaglandins mediated the induction of vitellogenesis in crabs *O. senex senex* by stimulating MF synthesis and consequent ecdysteroid production, and suggested that this phenomena is likely due to the inhibition of the release of MOIH and MIH from eyestalks, or based on the direct action of prostaglandins on the mandibular organs and/or the Y-organs.

Research on the intermediates of MF biosynthetic pathways have revealed the stimulatory effects of farnesoic acid (FA), the precursor of MF, on stimulating vitellogenin gene expression in hepatopancreatic and/or ovarian explants in the red crab *C. feriatus* (135), American lobster *H. americanus* (136) and penaeid shrimps *Metapenaeus ensis* (138) and *P. monodon* (139). These authors reported FA to be more potent than MF in stimulating vitellogenesis. FAMeT levels were also demonstrated to be positively correlated with ovarian development in *P. indicus* (29) and *P. monodon* (140). Serotonin-stimulated ovarian maturation was associated with an increase in hemolymph MF levels in *F. merguiensis* (51), as well as the expression levels of retinoid-X receptors in crab (69) and also with a significant increase in FAMeT levels in the mud crab, *S. olivacea* (47), *P. monodon* (140), and *P. indicus* (29). Furthermore, higher levels of FAMeT were observed in ablated *P. indicus* treated with serotonin (29), suggesting that serotonin-mediated stimulation of vitellogenesis occurs by stimulating MF synthesis. In the shrimp, *M. ensis*, the observed co-localization of FAMeT with CHH and MIH in the neurosecretory cells together with the comparatively higher transcript levels of FAMeT, CHH, and MIH proteins supports the notion of a possible interaction between eyestalk neuropeptides and FAMeT (141). These lines of evidence taken overall, indicate a synergistic interaction among the serotonergic, neuropeptide and MF pathways in regulating crustacean ovarian maturation.

### MF Signaling Pathways

A deeper understanding of the signaling pathways mediated by MF is necessitated in order to analyze the functional aspects of MF-regulated ovarian development in decapod Crustacea. However, many facets of this system remain unclear. For example, the characteristics of the MF receptor are unknown. In more detail, MF is thought to induce maturation through a mechanism based on ecdysteroid synthesis in the Y-organs. In this situation, MF acts as a ligand for retinoid-X receptors in synergy with ecdysteroids to stimulate the RXR–EcR heterodimer complex, initiating the expression of combined regulatory genes (E75 and E74) for both of these hormones in the hepatopancreas; this in turn induces vitellogenin gene expression (120, 142). Identification of two different RXRs in the ovary of green crab *C. maenas* and their expression levels at different vitellogenic stages indicates a role for retinoid-X receptors in crustacean ovarian development; this was further confirmed by experiments employing dsRNA-mediated silencing of retinoid-X receptors which lowered vitellogenic activity in this species.

MF can bind to membrane receptors and activate protein kinase C (PKC), and cause the subsequent modulation of potassium and calcium ion channels, resulting in a signal-transduction cascade that promotes vitellogenin uptake into the oocytes (143, 144). PKC is also well known to cause the activation of nuclear factor-kB (NF-kB) (145) and the mitogen-activated protein kinase pathway (MAP kinase) (146). On the contrary, the Ca^2+^/PKC signaling cascade was reported to suppress vitellogenesis in marine penaeid shrimps, where a signaling cascade (GPCR-receptor tyrosine kinase (RTK)-phospholipase C (PLC)-inositol trisphosphate receptor (IP3R)-PKC), similar to JH-induced cascades reported in insects such as the locust *Locusta migratoria* (147), was proposed to regulate vitellogenesis (148, 149). In *L. vannamei*, Alnawafleh et al. (32) provided evidence of the involvement of a Ca^2+^/PKC signaling cascade in vitellogenesis. On the contrary, Chen et al. (150) reported that PKC-α isoform may regulate ovarian growth in *L. vannamei* through a negative-based regulating mechanism. The functioning of Ca^2+^ signaling in ovarian development reported in different species is inconsistent, with strong evidence for fundamentally opposite (inducing and hindering) functions being attributed to it (15, 143, 148, 150, 151). These discrepancies relating to the actions of PKC in Crustacea appear to vary with the habitat of the species. Future experiments using PKC activators and inhibitors or CRISPR-mediated studies harbor much potential to shed light on the role of PKC in crustacean vitellogenesis.

## Vitellogenesis-Stimulating Hormone (VSH)

The brain and thoracic ganglia have been suggested to release a putative vitellogenesis-stimulating hormone (VSH) that promotes vitellogenesis and ovarian development (1, 25, 152). Though its identity is still unclear, in early research, it was suggested to be a peptide that can be inactivated by trypsin (153). Some workers have proposed it to be an analog of GnRH, and would similarly be released from the nervous tissue to stimulate the release of a crustacean gonadotropin (154). The ability of implanted nervous tissue (brain and/or thoracic ganglion), or application of their extracts to stimulate ovarian development and enhance vitellogenesis, has been observed in different crustacean species; this ability seems be conditional upon the sourced animals being reproductively active (25, 152, 155). More specifically, for example, repeated implantation of the brain and thoracic ganglion into juvenile female freshwater field crab *Parathelphusa hydrodromus* increased oocyte size; however, effects were more pronounced in adult females (156). Yano et al. (25) suggested that the gonad-stimulating effects of brain and thoracic ganglion are not species-specific, as ovarian maturation was accelerated in *L. vannamei* by the implantation of lobster ganglion.

Several neuromediators in crustacean reproduction, including serotonin (27) and RPCH (103), and in addition the juvenoid substance MF (29) are reported to indirectly exert their stimulatory effects by inducing the release of putative VSH from the thoracic ganglion. In *M. rosenbergii*, serotonin-primed thoracic ganglion medium stimulated oocyte growth and ovarian maturation (46). Furthermore, *in vitro* and *in vivo* studies have demonstrated that the release of putative gonad-stimulating factors from the brain and thoracic ganglia could be hampered by the presence of copper and cadmium (157), while stimulated by the application of calcium ionophore (A23187) (104). However, whether the proposed VSH functions directly or indirectly is yet to be conclusively confirmed. Although there are many studies where VIH has been extracted and chemically characterized based on the extensive collection of sinus glands, such as that has been accomplished by Tsutsui et al. in *L. vannamei* (158), the equivalent type of experimentation remains difficult with respect to isolating and characterizing crustacean VSH. This is because the concept of a putative VSH is tied to the existence of different stage-specific factors that stimulate vitellogenesis; or alternatively, a situation where an actual substance does not actually exist, but rather is based on a negative feedback mechanism to which the XO/SG complex is central (159). Thus, identifying and characterizing VSH in crustaceans remains a challenging research topic, the outcome of which could be of immense use in controlling the maturation of shrimp and other commercially-useful crustacean species in captivity, thus having important implications for the further development of the aquaculture industry.

## Potential Involvement of Other Factors (Ecdysteroids/Vertebrate Steroids, Gonadotropin-Releasing Hormone, Kisspeptin); Future Implications for Commercial Aquaculture

Despite the significant economic importance of many decapod crustacean species, overcoming the problem of reproductive dysfunction seen in captive broodstock remains a major bottleneck in crustacean aquaculture. Understanding the dynamics of the complex crustacean neuroendocrine system and the precise interactions among multiple neuromediators and various hormonal substances having pleiotropic functional roles would be useful in this regard. Nevertheless, this topic remains challenging, although many studies have been carried out employing immunohistochemical, molecular, and biochemical methods. It is, however, clear that neuropeptides from the eyestalks functionally regulate other mediators/hormones, especially those originating from the brain and thoracic ganglion, achieving a coordinated regulation of ovarian maturation. Among these neuropeptides, eyestalk peptides of the CHH-family have been reviewed extensively (10) in context of their roles in crustacean development and reproduction. In addition to the eyestalk peptides, diverse factors including non-eyestalk peptide hormones, steroid hormones and neurotransmitters, as well as their receptors, have been shown to regulate ovarian development in various crustacean species (160).

Subramoniam (31, 160) has reviewed the role of ecdysteroids in crustaceans, and has put forth that after being synthesized at the Y-organs, they are accumulated in developing ovaries perhaps based on a mechanism that involves binding with vitellogenin; such ecdysteroids are then thought to serve as a reserve for use during embryogenesis (by the developing embryo enclosed in eggs brooded externally). Ecdysteroids are therefore considered to serve as important hormonal factors that are involved in not only molting, but also in reproduction, drawing similarities to certain species of insects. In this way, molting and reproduction are inextricably linked, but each species exhibits its own unique pattern of growth and reproductive strategy.

The presence of vertebrate-type steroid hormones (estradiol, testosterone, pregnenolone and progesterone) has been revealed in several crustacean species [reviewed by Subramoniam (160)], and a correlative fluctuation of these substances in the hemolymph, hepatopancreas, and ovaries with the reproductive cycle has suggested a regulatory function in reproduction analogous to that in vertebrates (31, 161, 162). Nevertheless, *in vitro* and *in vivo* studies employing the direct administration of these hormones in crustacean species has yielded contrasting results, ranging from the absence of effects (163) to positive regulation of vitellogenesis and ovarian maturation (26, 164–166). A possible explanation for the above discrepancies may be related to the ovarian stage-specific and dose-dependent effects of the examined substances. Merlin et al. (26) reported hormone levels in hemolymph to be higher in ablated shrimps, suggesting the influence of VIH on the synthesis and the release of the sex hormones. Coccia et al. (165) demonstrated E2 to be more effective than progesterone in stimulating Vg mRNA synthesis in hepatopancreas. The existence of sex steroids receptors is controversial in crustaceans, although estrogen and progesterone receptors have been identified in various crustacean species (167, 168).

Furthermore, recent reports on the existence of gonadotropin-releasing hormone (GnRH)/gonadotropin secretion, kisspeptins and vertebrate-like steroids in crustaceans have provided new vistas for exploring the presence of an evolutionarily-conserved neuroendocrine signaling mechanism in crustacean reproduction, similarly to vertebrates, with a gonadotropin-releasing hormone (GnRH)/gonadotropin-like axis regulated by kisspeptin-related factors. Gonad development/maturation and male-specific hormone production in the androgenic gland were stimulated by the administration of exogenous GnRH, suggesting the possible existence of a GnRH-mediated regulatory mechanism comparable to that in vertebrates (54, 70, 169, 170). Additionally, the existence of receptors for GnRH (GnRHR) was also reported in the ovaries of *M. nipponense* (170) and Chinese mitten crab, *E. sinensis* (171). The presence of gonadotropin-like substances in crustaceans as described by Ye et al. (172, 173) further provide evidence for the existence of GnRH/GtH-mediated regulation in the reproductive process.

The presence of kisspeptin-like peptides and their receptors was reported in the neural tissues of *M. rosenbergii* by Thongbuakaew et al. (174); the co-localization of Kiss-I and GnRH signals in the same neurons suggested that locally-synthesized kisspeptin plays a pivotal role in ovarian maturation and spawning by exerting autocrine and paracrine regulation on GnRH secretion from neural and ovarian tissue. Moreover, injection of exogenous kisspeptin was also shown to induce ovarian maturation and spawning in *M. rosenbergii* perhaps via action on GnRH or stimulation of E2 production in the ovary.

Returning to the main factors covered in this review, *in vitro* and *in vivo* experiments together with immunolocalization studies have demonstrated that serotonin and RPCH function as neurohormones and neurotransmitters, respectively, and exert receptor-mediated effects on vitellogenesis and oocyte maturation through an autocrine/paracrine regulatory mechanism. Moreover, serotonin has been suggested to exert its downstream effects on reproduction through RPCH in several crustacean species. In addition, the correlation of circulatory levels of MF and the intermediates in its biosynthetic pathway with vitellogenin gene expression and ovarian development, suggests a direct regulatory role in vitellogenesis via the hepatopancreas, or an indirect role through the stimulation of the synthesis of other biological molecules (such as ecdysteroids from the Y-organs). Serotonin treatment may also trigger a significant increase in circulatory MF levels together with retinoid-X receptors expression in the hepatopancreas and ovary in crustaceans; RPCH may also stimulate MF synthesis in mandibular organs. Taken together, it is tempting to speculate that biogenic amine signaling, either alone or in combination with other factors including RPCH and MF, can potentially trigger the eyestalk neuropeptide signaling pathways to inhibit or stimulate the onset of ovarian maturation in an autocrine and/or paracrine manner.

Concurrently, with the recent advances in bioinformatic analysis of genomes and transcriptome and methods of mass spectrometry, our understanding of the functional roles of hormones/ neurotransmitters, other novel factors, and putative receptors involved in the complex regulatory network of reproduction has rapidly expanded. Of interest, recently much research attempting to elucidate the biological functioning of the various substances discussed in this review has been carried out based on approaches such as the application of recombinant or synthetic proteins and antibodies or utilization of dsRNAi/microRNAi techniques. Furthermore, RNAi harbors the potential to develop an artificial means of suppressing circulatory VIH levels, thus providing a partial replacement to eyestalk ablation currently used in hatcheries world-wide. For example, the reader is referred to Kang et al. (18) in which transcriptional silencing of VIH was achieved in *L. vannamei*. Gene-editing methodology may also yield new perspectives on the functional significance of various hormones in crustacean reproduction. Exploration of the possible use of gene-editing technologies such as CRISPR-Cas9 knock-ins or knock-outs will facilitate the acquisition of new knowledge on the regulatory roles of many neuromediators and other factors.

**Figure 5:**
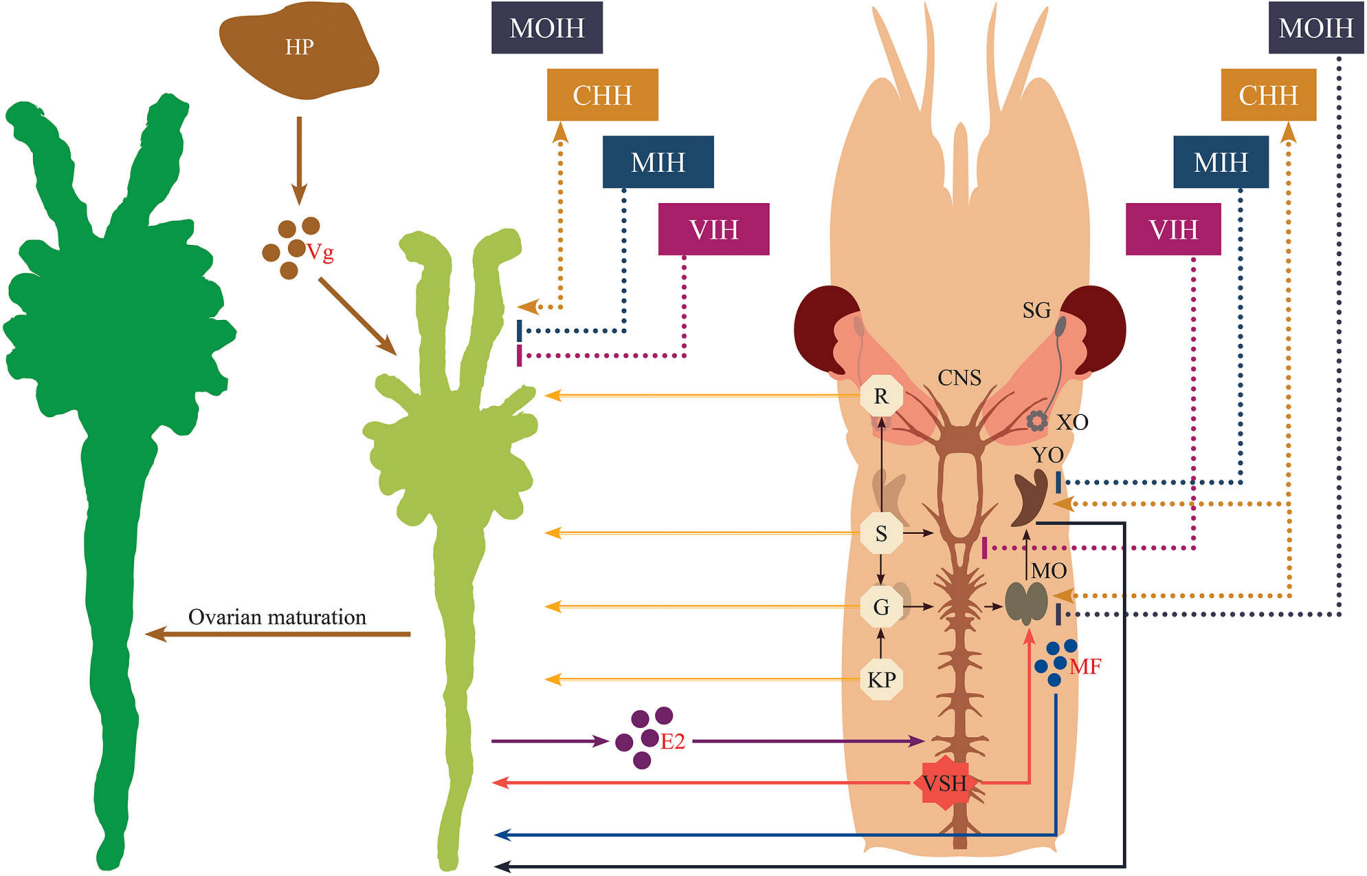
Schematic representation of the endocrine regulation of crustacean maturation. Nervous tissue is diagrammatically depicted for the cephalothoracic region of a typical shrimp. Stimulatory effects are indicated by lines ending in arrowheads, while inhibitory effects are indicated by lines having blunted ends. Neuropeptides originating from the eyestalk are displayed with dashed lines. Solid lines correspond to all other factors or actions. The secretory products from hepatopancreas, ovaries and mandibular organs are represented as colored circles with their abbreviations (vitellogenin, Vg; 17β-estradiol, E2, and methyl farnesoate, MF, respectively). Other abbreviations shown in the diagram are given as follows. SG, sinus gland; XO, X-organ; CHH, crustacean hyperglycemic hormone; MIH, molt-inhibiting hormone; VIH, vitellogenesis-inhibiting hormone; MOIH, mandibular organ-inhibiting hormone; VSH, vitellogenesis-stimulating hormone; YO, Y-organ; MO, mandibular organ; R, red pigment-concentrating hormone; S, serotonin; G, gonadotropin-releasing hormone; KP, kisspeptin; HP, hepatopancreas.

In conclusion, several hormones, neuromodulators and novel factors involved in the regulation of vitellogenesis are well-recognized; however, the cross-talk between them as well as their overlapping and multiple functions remain unclear. Another limitation is that information currently available on the functional roles of many neuromediators draws from research work performed on differing crustacean species; as noted above, functionality of hormonal substances and their related mechanisms in terms of the stimulatory aspects of reproductive regulation may vary widely. This is why, until the present, it is still difficult to develop a comprehensive framework that fully explains crustacean reproduction in the way that this has been accomplished for vertebrates. Nevertheless, in recent years, a great deal of knowledge has accumulated regarding the stimulatory aspects of crustacean reproductive development that go much beyond the simple scheme presented in Figure 1. Our understanding of the synergistic effects of distinct regulatory signaling pathways involved in crustacean ovarian maturation has increased in recent years, and here we have made an attempt to integrate this previously-established knowledge and latest advances into a new schematic diagram (Figure 5). As delineated in the figure, analogously to the case of vertebrates, the ovaries may release sex steroids which are proposed to exert a stage-specific regulatory feedback along the nervous-gonadotropic axis.

The underlying mechanisms by which multifunctional neuropeptides, neurotransmitters/neuromediators and other factors switch their functional roles and act in concert together with other regulatory pathways merits further investigation. Such studies are expected to provide greater insight into reproductive mechanisms, and harbor potential applications in the development of maturation techniques that may be employed in crustacean aquaculture.

## Author Contributions

VJ: conceptualization and writing. ST: writing and data/manuscript curation. MNW: writing and editing.

## Conflict of Interest

The authors declare that this manuscript was prepared in the absence of any commercial or financial relationships that could be construed as a potential conflict of interest.
